# How do digital techniques of shape capture and alignment compare to traditional casting methods when applied to pediatric ankle-foot orthoses (AFOs)?

**DOI:** 10.1371/journal.pone.0331895

**Published:** 2025-09-10

**Authors:** Connor Matton, Calvin C. Ngan, Jan Andrysek

**Affiliations:** 1 Institute of Biomedical Engineering, University of Toronto, Toronto, Ontario, Canada; 2 Bloorview Research Institute, Holland Bloorview Kids Rehabilitation Hospital, Toronto, Ontario, Canada; Chongqing University Three Gorges Hospital, CHINA

## Abstract

Achieving optimal alignment and fit is a key aspect of ankle-foot orthosis (AFO) design, as it directly influences the effectiveness of the device. While digital workflows offer the potential to integrate quantifiable alignment measures and corrections into AFO design, a major challenge remains in controlling lower-limb positioning and alignment during 3D scanning. This study aimed to evaluate pediatric AFO alignment and shape differences of directly scanned (live scan) vs casted lower limb models. Eighteen participants aged 4−16 years treated by 5 certified orthotists were recruited. Participants and casts were scanned. Sagittal plane ankle-foot alignment differences were analyzed between pairs of live scan and cast models. Using digital tools, the ankle-foot alignment of the live scans was then corrected, and the alignment differences were re-evaluated to assess the re-alignment methods and allow for further shape comparisons. After correction, modification maps were generated to assess the shape differences (surface deviations) between the live scans and cast models. Shape differences were also assessed with respect to participant characteristics. The results of this study demonstrated that AFO users can be scanned in a nearly corrected position (mean sagittal plane angle difference = 0.85°, SD = 4.44°), and that digital tools can be used to measure and adjust ankle-foot alignment with high accuracy (<1°error). The modification maps revealed that the live scans closely matched the cast models, with shape differences consistently observed in the foot and heel regions. Mean differences ranged −2.12–1.45 mm, positive differences (cast larger) ranged 1.14–2.71 mm, and negative differences (cast smaller) ranged 1.50–3.47 mm. Height, age, and foot length had moderate effects on shape differences (ρ = 0.5–0.75), while significant differences were observed between orthotists (∊^2 ^= 0.32). These findings can drive future advancements in the digital design and fabrication of AFOs.

## Introduction

Ankle-foot deformities can affect gait and contribute to increased pain and discomfort during activities of daily living. These issues can lead to a range of complications, including chronic pain, limited mobility, joint damage, muscle imbalances, and heightened risk of falls, all of which can significantly impact an individual’s daily functioning and overall quality of life [1]. Custom-made ankle-foot orthoses (AFOs) are commonly utilized in patient care and rehabilitation to improve and restore proper ankle-foot alignment, support, mobility, and stability [2–4]. When designing an AFO, orthotists must consider the patient’s functional goals, assess their anatomy, select appropriate materials, and ensure a comfortable fit [5]. Achieving optimal alignment and fit is crucial, as it directly influences the AFO’s effectiveness in improving gait, enhancing stability, and correcting ankle-foot deformities [6].

Both traditional (casting and moulding) and digital techniques (scanning, CAD/CAM) depend heavily on the orthotist’s expertise during shape capture, design, and fabrication [7]. While casting inherently involves human error and material variability, it has demonstrated favourable clinical outcomes, establishing it as a benchmark for comparison in evaluating 3D scanning for AFOs [8,9]. Digital technologies (DT) offer the potential to reduce manual labor, facilitate easier storage and modification of models, and provide greater design flexibility [10]. However, integrating DT into AFO design and fabrication presents challenges, particularly during shape capture. These challenges can introduce variability in foot parameters such as alignment and morphology which affect fit and function [8,11].

Much of previous research has focused on the accuracy and reliability of scanning the lower-leg, ankle, and foot morphology in able-bodied individuals rather than AFO users [12]. Special considerations are necessary for AFO users with underlying neuromuscular and musculoskeletal conditions that can impact scan quality [13]. Involuntary movements, due for example to spasticity and ankle-foot deformities caused by various pathologies, can influence the scanning process. These issues may lead to misalignment and artifacts in the scan data [14,15]. Additionally, individuals with conditions such as autism spectrum disorder (ASD) may experience difficulty cooperating or may feel apprehensive about using DT. Shape capture results can also differ significantly between weight-bearing and non-weight-bearing scans [11].

Foot morphology is primarily influenced by the alignment of the ankle joint and foot with sagittal plane alignment being critical to the biomechanical effectiveness of an AFO [16,17]. While the frontal and transverse planes are also important, they can be particularly challenging for certain clinical populations such as cerebral palsy and spina bifida [18,19]. Sagittal-plane alignment is sometimes adjusted after limb capture, either through plaster modification or digitally using CAD; however, this can introduce other challenges and extra work [6,9]. Therefore, achieving proper alignment during the initial shape capture process is crucial. Despite this, the processes for AFOs are not well established, resulting in a considerable variability in outcomes in terms of the captured shape [11]. Furthermore, measuring ankle-foot alignment is not common clinical practice, as it can be time-consuming and requires specialized equipment [20]. Consequently, there is little consensus on how to measure and correct alignment, what constitutes ideal alignment for specific patients, or what outcomes are considered acceptable.

The overall aim of this study was to evaluate a 3D scanning process for AFO design, including both limb positioning and digital shape capture. Specifically, the study compared sagittal-plane ankle-foot alignment of direct scans of the lower limb (live scan) and cast models. Alignment differences between the live scans and cast models were assessed as originally scanned, and also once adjusted/corrected using DT CAD tools. A further aim was to assess the global and local shape differences between these pairs and their relationship with participant characteristics (height, weight, age, gender, foot length, orthotist, primary diagnosis and AFO type). To our knowledge this is one of the first studies to compare digital shape captures and casted models of pediatric AFOs.

## Methods

### Shape capture process

#### Setup.

Two primary scanning methods were considered: weight bearing and non-weight bearing. Weight bearing scans often use an apparatus to support the patient’s lower limb during scanning. However, orthotists lose tactile control over limb positioning, which complicates alignment corrections [12,21]. In non-weight bearing scans, where the patient is seated or lying down, the orthotist can maintain tactile control of the lower limb. The orthotist’s hands may interfere with the scan and introduce artifacts, although these can practically be removed from the scan data during model post-processing [22,23].

**Fig 1:**
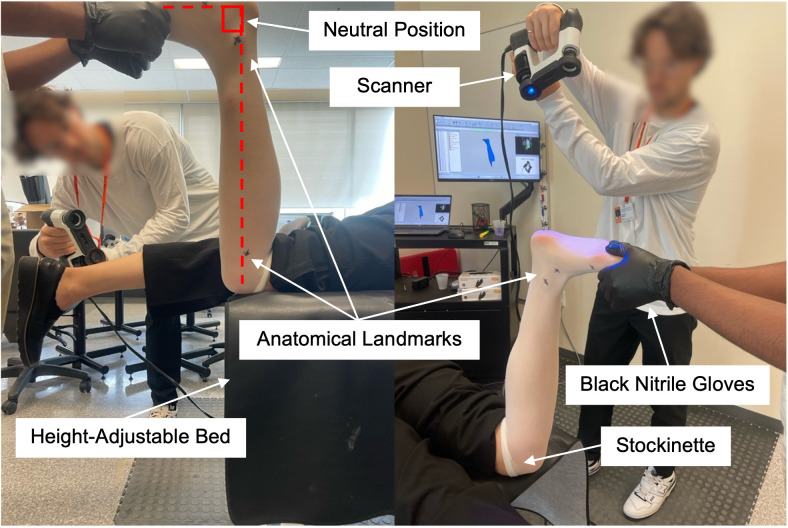
Scanning protocol for digital shape capture. The scanning protocol showing the participant (on the height adjustable bed), the orthotist (holding the participant’s foot), and the trained expert performing the scan. The orthotist ensured the participant’s knees aligned with the bed’s end and adjusted their ankle-foot alignment by bending the knee and raising the foot. After adjusting and maintaining the participant’s foot position with their hands held distal to the metatarsal heads, the orthotist and the participant were instructed to remain still for the duration of the scan.

As part of the pilot work, we collaborated with orthotists from the Orthotics and Prosthetics (O&P) department at Holland Bloorview Kids Rehabilitation Hospital (Toronto, ON, Canada) to design and evaluate the scanning methods described. The goal was to scan the limb in a nearly corrected position. Non-weight-bearing, prone positioning was found to provide optimal control over ankle-foot alignment, making it suitable for most patients and was therefore chosen for the study (Fig 1). A height-adjustable bed on which the patient lay allowed the orthotist to maintain a comfortable upright posture—standing with a straight back, relaxed shoulders, and elbows bent at 90 degrees—while holding the patient’s foot as another individual performed the scan. The orthotist wore black nitrile disposable gloves to enhance color contrast between their hands and the patient’s limb, reducing background noise and minimizing artifacts in the scan data. Scanning was performed by a second individual, in this case researcher and co-author M.C., who had been trained to operate the instruments.

#### Instruments.

The Spectra scanner by Vorum (Vancouver, BC, Canada) was used to scan the participant’s limbs and casts [24]. This handheld 3D structured light scanner operates at a resolution of 0.1 mm and a working distance of 35–50 cm, capturing 15 photographs per second. The Spectra software (Vorum, Vancouver, BC, Canada) was used to generate real-time 3D models from the scans.

#### Shape capture procedure.

Prior to the digital shape capture, the orthotist performed a comprehensive physical assessment of the limb, evaluating skin integrity, muscle strength, joint range of motion (ROM), and joint stability. To ensure consistent skin tone during the scan, a stockinette was placed over the participant’s limb. Anatomical landmarks and bony prominences were then marked on the stockinette using an indelible pen (Fig 1). These landmarks included the fibular head, medial and lateral malleoli, navicular, base of the 5th metatarsal, 1st and 5th metatarsal heads, and calcaneus. In traditional rectification, areas surrounding bony prominences require plaster build-ups to create additional space for comfort and to reduce pressure between the limb and the interior surface of the device [25]. The indelible pen markings help guide orthotists during traditional rectification, ensuring an optimal fit and minimizing the need for adjustments [26].

The participant was then instructed to lie prone on the height-adjustable bed, and the orthotist adjusted their position to achieve the target ankle-foot alignment. In clinical practice, the neutral angle describes the sagittal plane alignment of an AFO, with benchmark alignment typically set at 90 degrees (Fig 1) [6,27]. However, to allow for a direct comparison between traditional and digital shape capture, the orthotist targeted the same alignment as the participant’s cast. The orthotist maintained this position while the trained expert performed the digital scan. Starting at the medial malleoli, the scanner was moved continuously around the leg to capture the medial, anterior, and posterior surfaces. The scanner was then elevated to capture the plantar surface, with the dorsum of the foot scanned last. Moving around the orthotist to the lateral side, scanning continued along the posterior leg, following the same pathway used on the medial side. Once the scan was completed, the orthotist reviewed the results to ensure that: (1) all anatomical landmarks had been captured and (2) they were satisfied with the ankle-foot alignment, otherwise the scan was repeated. Each scan took between 30 to 90s.

### Data collection

#### Participants.

Between October 1st, 2023, and May 1st, 2024, a convenience sample of participants was recruited in collaboration with orthotists from the O&P department at the Holland Bloorview Kids Rehabilitation Hospital (Toronto, ON, Canada). Orthotists were certified with a minimum of 5 years of clinical experience in AFO design. Children and youth aged 4–16 years who were prescribed an AFO at the hospital were eligible for inclusion if they could 1) transfer independently or with assistance to a plinth 2) listen to and follow instructions as judged by the treating orthotist 3) be taken into a subtalar neutral position. For children with a bilateral prescription, one side was chosen at random. Participant characteristics such as primary diagnosis, height, weight, age, gender, and foot length were recorded, along with the type of AFO prescribed. Approval for the study was granted by the Research Ethics Board at Holland Bloorview Kids Rehabilitation Hospital (REB # 0253) (Toronto, ON, Canada) and written consent was provided by each participant’s parent or guardian.

#### Traditional and digital shape capture.

Both the traditional and digital shape capture methods were performed in the same session, enabling a direct comparison between the live and cast models. First, ankle-foot measurements were taken and recorded by the treating orthotist. Traditional shape capture was performed first using 3M™ Scotchcast™ Plus Casting Tape. During this process, the orthotist wrapped the casting tape around the participant’s ankle, foot, and lower leg in a non-weight bearing position. While the tape hardened, the orthotist held the patient in optimal alignment (subtalar neutral position) by applying pressure to key areas of the ankle and foot while maintaining the lower limb in a suspended, non-weight bearing position. By applying pressure, the orthotist could control the subtalar joint, an articulation between the talus and calcaneus responsible for supination and pronation of the foot—movements that occur across all three anatomical planes [28]. Once the tape set, the scotch cast was removed. This process is a well-established practice used by orthotists at Holland Bloorview Kids Rehabilitation Hospital during AFO fabrication. Following the traditional method, the limb was scanned by the trained expert (M.C.) with the help of the treating orthotist. The scanning technician had completed about 5 h of formal training (provided by Vorum) and had experience with the scanner and scanning protocols prior to data collection. The orthotist aimed to adjust and maintain the positioning of the participant’s lower limb to match the alignment of the cast for the duration of the scan. After the session, the scotch cast was filled with plaster to create a positive ‘unmodified’ cast. This cast was held in a stand by a mandrel while it was scanned by M.C. to create the digital model. The orthotists also completed a short evaluation survey following the session to provide feedback on their experiences with both the traditional and digital shape capture processes.

### Data processing

#### Coordinate system.

**Fig 2:**
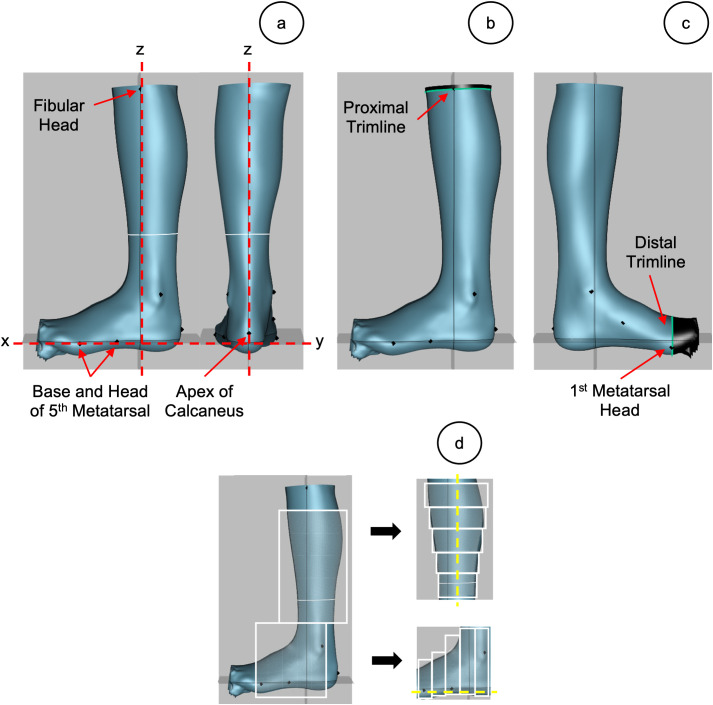
Orienting the AFO models using an XYZ coordinate system. a) Anatomical landmarks were used to define the XYZ coordinate system (red axes). The X-axis is defined by the base and head of the 1st metatarsal, while the Z-axis intersects through the fibular head and is orthogonal to the X-axis. The XZ-plane aligns with the apex of the calcaneus and the Y-axis is orthogonal to both the X- and Z-axes. b) The proximal trimline intersected the fibular head in the XY-plane. c) The distal trimline intersected the 1st metatarsal head in the YZ-plane. d) Point-cloud cross sections are used to create 2 vectors (yellow axes), which represent the plantar surface of the foot and the tibia.

Spectra and Canfit O&P CAD software (Vorum, Vancouver, BC, Canada) was used to post-process the digital models. The integration of Spectra with Canfit provides clinicians with a fully digital workflow that can be adopted into clinical practice [29]. The anatomical landmarks were identified on the digital models from the scanned images. Using these landmarks, a coordinate system was developed to orient the models before measuring sagittal plane alignment. The sagittal plane alignment of an AFO was defined as the position of the shank relative to the plantar surface, represented by the angle between two reference lines that correspond to each [27]. This method was adopted in the digital process by using the anatomical landmarks to define the XYZ coordinate system (Fig 2a). The orientation of the models was critical for measuring sagittal plane alignment and ensuring the foot and shank were aligned with the x and z axes respectively. Models were then trimmed based on commonly used trimlines used in clinical practice (Fig 2b and c) [30].

#### Ankle-foot alignment algorithm.

Sagittal plane alignment was calculated digitally using a previously developed Matlab algorithm (The MathWorks, Inc., Natick, MA, USA) [31]. All of the digital models were first exported from Canfit to CloudCompare software (2.13) as “stl” files. Point-cloud cross-sections of the foot and tibia were created along the z and x axes, respectively. Each of these larger cross-sections was further divided into 5 equal segments (Fig 2d) [31]. These point-clouds were then converted into “txt” files to be processed in Matlab. The algorithm uses the indices of the minimum z-values in each of the five sections of the foot. The corresponding x, y, z coordinates at these indices are extracted, resulting in five 3D points. A linear regression was applied to create a line of best fit. A similar process was applied for the tibia resulting in 2 vectors representing the plantar surface of the foot and tibia (Fig 2d). The 2D angle between the vectors defined the sagittal plane alignment of the model.

#### Corrected/adjusted AFO alignment.

**Fig 3:**
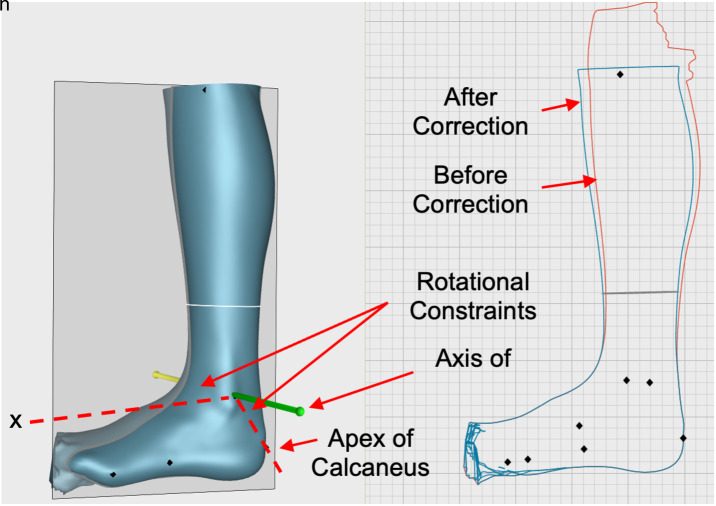
Canfit constrained rotate tool. The Constrained Rotate tool in Canfit was used to adjust ankle-foot alignment. The rotational constraints were set through the apex of the calcaneus and along the X-axis to ensure that only the leg was being bent. The shape of the foot was preserved, and ankle-foot alignment was corrected by bending the leg and moving the model into dorsiflexion or plantarflexion.

Ankle-foot alignment of the live scans was adjusted using the Constrained Rotate tool in Canfit to match the alignment (plantarflexion/dorsiflexion) of their corresponding casts (Fig 3). This adjustment enabled 1) the evaluation the Canfit tool for correcting ankle-foot alignment and 2) eliminating the effect of sagittal plane alignment when comparing the localized ankle/shank shape differences, since without this adjustment, mesh registration would result in a misalignment of the foot and shank sections of the models. The axis of rotation was defined through the malleoli, based on how the ankle bends biomechanically and giving leverage over the traditional wedging process [28]. Alignment corrections were made to the nearest degree based on the previously calculated differences. Adjustments were made only for the live scans with differences exceeding one degree. This ‘one degrees’ target is significantly more stringent than those typically used by orthotists when correcting scotch casts that are not optimally aligned [6].

#### Mesh registration.

Mesh registration was conducted using Matlab and CloudCompare to align and overlay the 3D models of the live scans and the casts. Principal component analysis was performed in Matlab to preliminarily align the models. CloudCompare was then used to refine the registration via an iterative closest point algorithm.

### Data analysis

#### Alignment comparison.

To assess alignment differences, the live scans (uncorrected) and cast models were directly compared. To evaluate the ability of the Canfit tool to adjust sagittal-plane alignment, the alignments between the corrected live scan and casts models were compared.

#### Global shape differences.

Using Meshmixer 3.5 (Autodesk Inc., San Rafael, CA, USA), the volumetric measurement for each model was determined. The total volume variation was then calculated to evaluate the global shape differences between each pair of live and cast models. To further examine the global shape differences, the models were sliced in the X-Y plane along the Z-axis at intervals of 1% and the cross-section area (CSA) of each slice was computed using Matlab [32].

#### Localized shape differences.

A modification map was used to quantify and visualize the localized shape differences between live and cast models [29,33]. Each pair was compared in CloudCompare by their computed surface deviation [34]. The cloud-to-mesh difference (CMD) calculates the distance between corresponding (closest) points between the live and cast models. Positive depth values indicate areas that are larger (elevated) on the cast while negative values represent smaller (depressed) areas [25]. A method previously employed for characterizing localized shape differences in prosthetic sockets was used to create the modification map [29]. This method uses a threshold to identify significant positive and negative surface deviations. The positive and negative depth values are separated into two groups and the mean of each group is calculated and used as a threshold. Depth values exceeding this threshold are represented visually on a modification map. For this study, a gradient two-colour scheme was projected onto the cast to visualize positive and negative changes. These threshold values were also be represented numerically by the mean value of CMD (mCMD), the average of the positive values of CMD (pCMD), and the average of the negative values of CMD (nCMD) [33]. The modification maps were analyzed in collaboration with the study orthotists to identify trends and correlations between the models.

#### Intra-rater reliability.

An intra-rater reliability study previously used for AFO models was applied to assess the consistency of the registration process [33]. Ten pairs of live and cast models were randomly selected and registered twice using the same protocol. This sample size is adequate to detect an ICC of 0.9 with 80% power and a significance level of 0.05 [35]. The values of mCMD, maximum pCMD, and maximum nCMD were used to assess the reliability of the registration process.

#### Statistical analysis.

Statistical analysis was conducted using JASP (JASP Team, 2024). Non-parametric tests were used as the results of Shapiro-Wilk tests revealed that the data were not normally distributed. A paired two-tailed Wilcoxon signed-rank test was used to examine the differences in the 2D angle (sagittal plane alignment) between the live scan and cast models before and after correction [36]. Bland-Altman plots were created to assess the agreement between alignment measurements [37]. To evaluate registration reliability, intra-class correlation coefficients (ICC) were calculated using the ICC (3,1) equations with a 95% confidence interval (CI) [38]. Additionally, Kruskal-Wallis ANOVA was used to compare the means of cloud-to-mesh distances across orthotists, primary diagnosis and AFO type groups, with effect size measured using Epsilon squared, ∊^2^ [36,39]. The strength of the relationship between variables was assessed using Spearman’s correlation coefficient, ρ [40]. Welch’s t-test was used to compare the means in groups of prescription and gender, with effect size measured using Cohen’s d [36,39]. A significance level of α = 0.05 was used for all tests. The paired two-tailed Wilcoxon signed-rank test tested for differences in orthotists’ satisfaction scores between the digital and traditional shape capture methods.

## Results

### Participants

Twenty participants were recruited from 5 orthotists. Two participants were excluded due to challenges with scanning. Specifically, while moving around the orthotist to scan the posterior leg, the scanner had difficulty detecting the reference frame, leading to an incomplete scan. Ten participants required rescanning (done during the same session) because the orthotists were not satisfied with the results (alignment and shape) of the initial scan. 18 pairs of live and cast models were used for analysis. The participants ranged from 4–16 years old (10 males, 8 females). The mean age was 9.8, with a standard deviation of 3.5 years. Participants were divided into 3 groups based on their primary diagnosis: upper motor neuron (UMN, n = 8), lower motor neuron (LMN, n = 6), and ‘Others’ (n = 4). UMN lesions are present with hypertonia and spastic paralysis whereas LMN lesions were associated with hypotonia and flaccid paralysis [41]. Those with diplegic or hemiplegic cerebral palsy and stroke conditions were classified as ‘UMN’, while those with spina bifida and acquired brain injury were classified as ‘LMN’. Autism, idiopathic toe walking, and developmental delay conditions were classified as ‘Others’. Three types of AFOs were prescribed: hinged (n = 13), rigid (n = 3), and anterior ground reaction (AGR, n = 2). Prescription was either unilateral (n = 6) or bilateral (n = 12).

### Evaluating alignment differences

**Fig 4:**
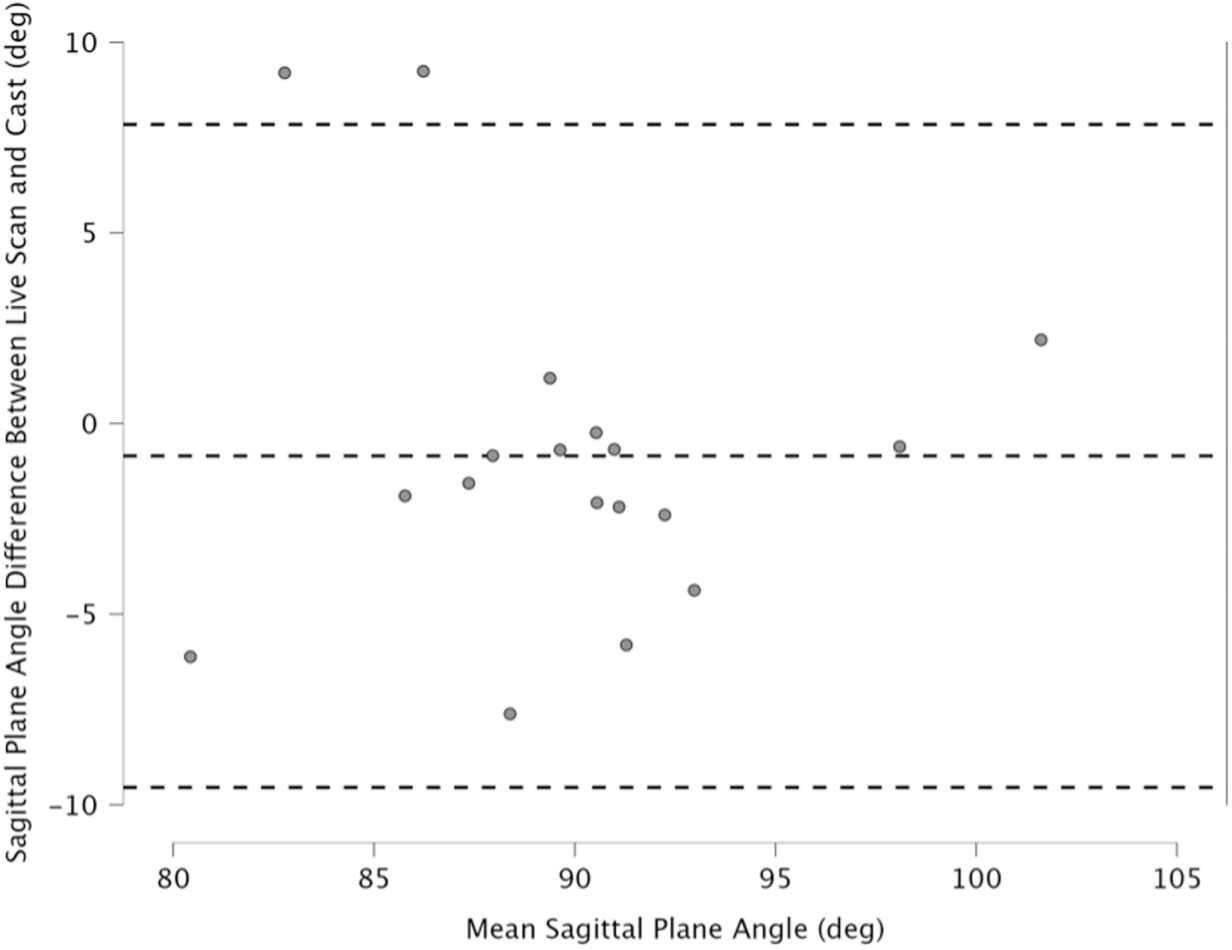
Bland Altman plot comparing sagittal plane angle of live and cast models before correction.

**Fig 5:**
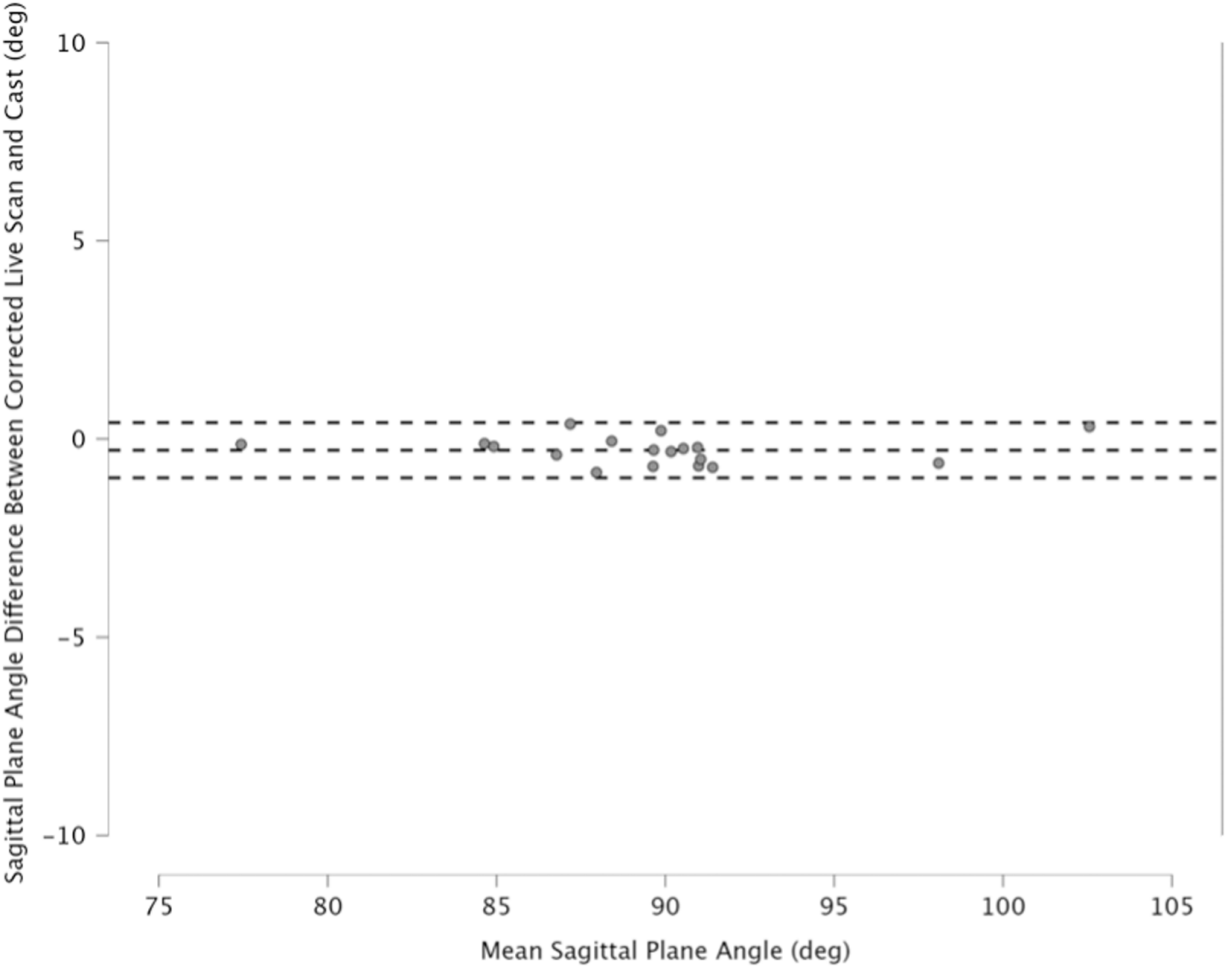
Bland Altman plot comparing sagittal plane angle of live and cast models after correction.

The pairwise comparisons between the (uncorrected) live and cast models revealed that 78% (n = 14) of the cast models were more dorsiflexed (Table 1). However, based on the paired two-tailed Wilcoxon signed-rank test, there was no significant difference in the sagittal plane alignment (p = 0.15). The mean sagittal plane angle difference between the live and casted models was slight dorsiflexion at −0.85° (range: −7.62° to 9.24°, SD = 4.44). Furthermore, 28% (n = 5) of the live scans were within the one-degree target of their corresponding cast; the remaining scans alignments were adjusted for the shape comparison. The paired two-tailed Wilcoxon signed-rank test showed a significant difference in the sagittal plane angles between the corrected live and cast models (p = 0.01). However, the mean sagittal plane angle difference was −0.28° (range: −0.85° to 0.38°, SD = 0.36). Bland-Altman plots comparing the sagittal plane angle differences before and after correction (Figs 4 and 5).

**Table 1:** Absolute alignment results and global volume differences for digital and traditional shape capture.

Participant	Cast Sagittal Plane Angle (deg)	Live Sagittal Plane Angle (deg)	Cast Volume (mm^3^ x 10^6^)	Live Scan Volume (mm^3^ x 10^6^)	Volume Difference Relative to Cast (%)
P1	90.01	92.20	1.44	1.48	−2
P2	90.41	90.65	1.13	1.17	−4
P3	87.37	78.18	1.21	1.30	−7
P4	89.51	91.59	1.26	1.41	−11
P5	97.79	98.40	9.95	9.65	3
P6	84.58	92.20	9.71	1.02	−5
P7	102.71	100.52	3.30	3.18	4
P8	91.04	93.44	3.25	3.16	3
P9	86.58	88.15	1.74	1.83	−5
P10	87.54	88.38	1.82	1.87	−3
P11	90.64	91.33	7.57	7.82	−3
P12	89.29	89.98	1.15	1.21	−5
P13	90.85	81.61	8.59	8.56	0
P14	77.37	83.49	1.08	1.16	−7
P15	89.98	88.79	5.33	5.48	−3
P16	88.38	94.19	1.97	2.04	−3
P17	84.82	86.72	1.39	1.40	−1
P18	90.79	95.17	1.86	1.89	−1
Mean (SD)	**90.28 (5.38)**	**89.43 (5.07)**	**1.44 (7.39)**	**1.52 (7.07)**	**−3 (4)**

For the volume difference data, negative values indicate cases where the live scan was larger, whereas positive values indicate cases where the cast was larger.

### Reliability of registration

The ICC_(3,1)_ values for mCMD, maximum pCMD, and maximum nCMD were all 1.000, indicating high reliability in the registration process [38]. Moreover, the mean percent errors were notably low: mCMD (0.87%, SD 1.43), maximum pCMD (0.17%, SD 0.25), and maximum nCMD (0.22%, SD 0.35), further confirming the strong reliability.

### Identifying global shape differences

The volume difference between each pair of live and cast models indicate a slightly larger overall volume of the live scans relative to casts (Table 1).

**Fig 6:**
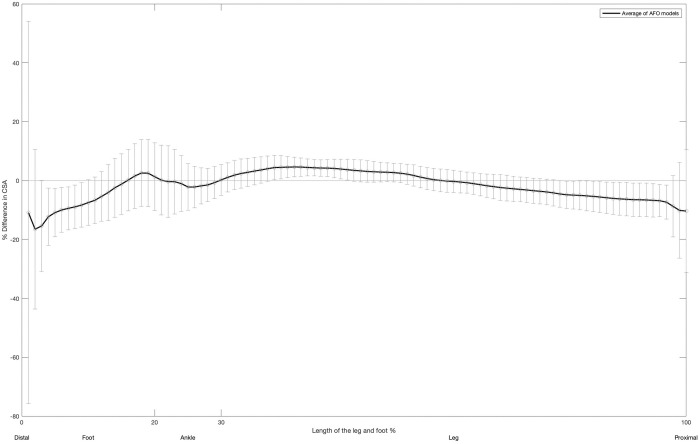
Mean differences in CSA between live and cast models along the length of the limb. Shaded bands represent one standard deviation. Positive values denote the mean increase in CSA in the cast model relative to the live model, while negative values indicate the mean reduction in CSA.

The differences in CSA between the live and cast models along the length of the limb are illustrated below (Fig 6). The graph shows a consistent trend across all pairs with negative differences in CSA (trendline below zero) observed at both the distal and proximal ends of the model. This suggests that the larger overall volume found in the live scan was primarily concentrated in these areas.

### Identifying localized shape differences

#### Modification map.

**Fig 7:**
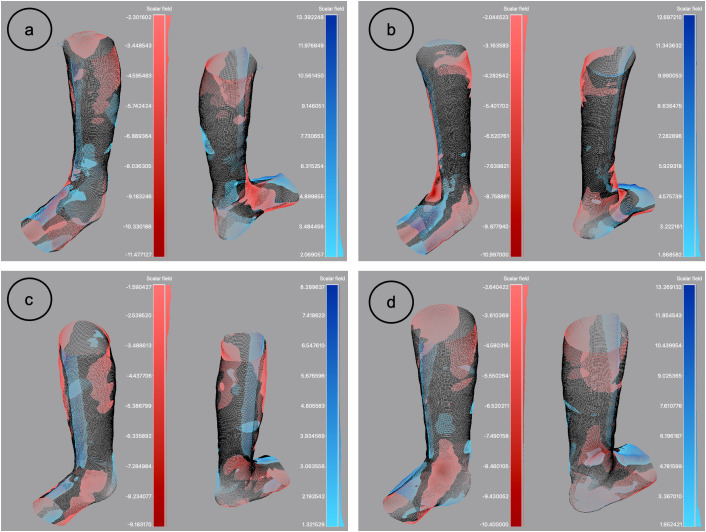
Anterior and posterior view of the modification maps of four participants. a) Participant categorized as “Others”, male, with bilateral condition, and prescribed a hinged AFO. b) Participant categorized as “UMN”, male, with bilateral condition, and prescribed a AGR AFO. c) Participant categorized as “LMN”, female, with unilateral condition, and prescribed a hinged AFO. d) Participant categorized as “UMN”, male, with unilateral condition, and prescribed a hinged AFO.Red indicates depressed areas, whereas blue indicates elevated areas of cast vs live.

The modification map illustrates the key areas of shape differences between the live scan and the cast, projected onto the cast (Fig 7). Depressed areas were frequently observed on the inner (medial) and outer (lateral) sides of the foot, as well as on the upper leg, whereas elevated areas were identified on the plantar surface, dorsum of the foot, and cut strip. The heel shows both depressed and elevated areas. Starting on the lateral side, depressed areas extend from the head to the base of the 5^th^ metatarsal and typically all the way to the heel, partially wrapping around its posterior and underside surfaces. On the medial side, depressed areas start at the head of the 1st metatarsal, extend into the medial arch, and continue up toward the navicular and medial malleoli. Elevated areas are also found on the medial side of the heel, again partially including its posterior and underside surfaces. One participant had the opposite pattern on the heel where depressed areas were found on the lateral side and elevated areas on the medial. In addition, another participant had a completely different pattern where depressed areas were only found in the upper leg. While trends in the locations of depressed and elevated areas were identified, the sizes of these areas varied among participants.

#### Cloud-to-mesh distance.

The mCMD, pCMD and nCMD of each pair of models were compared with participant height, weight, age, gender, foot length, orthotist, primary diagnosis and AFO type. Across all participants, the mCMD was −0.43 mm (range: −2.12 mm to 1.45 mm), the pCMD was 1.83 mm (range: 1.14 mm to 2.71 mm), and the nCMD was 2.26 mm (range: 1.57 mm to 3.47 mm).

Participant characteristics demonstrated varying relationships with the measures of CMD. Height exhibited a moderate direct relationship with mCMD (ρ = 0.57, p = 0.02), a fair direct relationship with pCMD (ρ = 0.28, p = 0.29), and no significant relationship with nCMD (ρ = −0.11, p = 0.67). Weight showed a fair direct relationship with mCMD (ρ = 0.32, p = 0.19) and pCMD (ρ = 0.35, p = 0.15), and no significant relationship with nCMD (ρ = 0.12, p = 0.64). Age revealed a moderate direct relationship with mCMD (ρ = 0.61, p = 0.01), while showing no significant relationships with pCMD (ρ = 0.21, p = 0.40) and nCMD (ρ = −0.24, p = 0.33). Lastly, foot length demonstrated a moderate direct relationship with mCMD (ρ = 0.66, p = 0.00) and pCMD (ρ = 0.46, p = 0.00), and no significant relationship with nCMD (ρ = −0.09, p = 0.73).

The analysis using one-way ANOVA did not reveal significant differences between AFO types when comparing mCMD (∊^2^ = −0.11, p = 0.87), pCMD (∊^2 ^= −0.03, p = 0.46), and nCMD (∊^2 ^= −0.00, p = 0.37). Similarly, there were no significant differences observed between primary diagnoses for mCMD (∊^2^ = 0.07, p = 0.22), pCMD (∊^2^ = 0.19, p = 0.09), and nCMD (∊^2^ = 0.06, p = 0.24). However, a significant difference was detected between orthotists for mCMD (∊^2 ^= 0.32, p = 0.05), while no significant differences were found for pCMD (∊^2^ = 0.00, p = 0.37) and nCMD (∊^2^ = −0.15, p = 0.95).

Welch’s t-test revealed no significant differences between gender groups for mCMD (d = 0.70, p = 0.18), pCMD (d = 0.35, p = 0.49), and nCMD (d = −0.06, p = 0.90). Additionally, no significant differences were observed between prescription groups for mCMD (d = −0.13, p = 0.79), pCMD (d = 0.08, p = 0.88), and nCMD (d = 0.28, p = 0.58).

#### Orthotists.

Orthotists rated their satisfaction with the time required, ease of the process, and results of both traditional and digital shape capture on a scale of 1–5, with 1 indicating “not satisfied at all” and 5 indicating “very satisfied”. The mean satisfaction scores were 4.44 (SD = 0.51), 4.33 (SD = 0.59), and 4.17 (SD = 0.62) for traditional shape capture and 4.06 (SD = 0.80), 4.00 (SD = 0.69), and 4.11 (SD = 0.58) for digital shape capture. When comparing the orthotists’ satisfaction scores for digital and traditional shape capture methods, a significant difference was observed in the time required (p = 0.03). However, no significant differences were found for ease of the process (p = 0.07) and results (p = 0.78).

## Discussion

This paper examined the differences in casting vs live scanning of the lower limbs, and to our knowledge is one of the first to do so on AFO-relevant populations. While studies have compared 3D scanning to traditional methods for capturing ankle-foot morphology, no attempts have been made to directly compare the shapes obtained from both methods [12,23,42]. Overall, the preliminary findings demonstrate that direct scans of lower limbs closely resemble cast models. Variability in lower limb scans primarily arises from limb positioning, alignment, and hand compressions during scanning. These findings aim to inform the future digital design of AFOs, supporting clinicians in developing fully integrated digital workflows.

Accurately aligning the lower limb during 3D scanning poses a significant barrier to the wider adoption of DT in designing and fabricating AFOs [8]. Although 3D scanning with custom jigs has proven effective in studies involving healthy individuals, their limited adjustability creates challenges with accurately aligning the lower-limbs of patients with severe ankle-foot deformities [12,21,23]. During the scan, orthotists are unable to stabilize and hold the patient’s foot and lower leg, which complicates the process of making alignment corrections [8]. In this study, scanning was performed in a non-weight bearing prone position, allowing orthotists to gain control over limb positioning. The orthotists visually targeted the same ankle-foot alignment as the participants’ casts, which was based on their individual capabilities and needs. Although orthotists typically aim for a 90-degree bend at the ankle, ankle-foot deformities restricting ROM can make this goal difficult to achieve [6,16]. The absolute alignment results confirmed that the orthotists were not able to achieve this alignment for all participants during traditional shape capture. While the mean sagittal plane angle of the casts was 90.28°, the standard deviation of 5.38 highlights the variability in ankle-foot alignment. Some participants were cast in plantarflexed positions as low as 77.4° and in dorsiflexed positions as high as 102.7°. Although differences were observed between the traditional and digital shape capture methods, the results of this study demonstrate that patients can be scanned in a nearly corrected position, and that sagittal ankle-foot alignment can be accurately measured and adjusted quantitatively using CAD tools. Specifically, the mean sagittal plane angle difference between the live and cast models was −0.85° with a standard deviation of 4.44. These alignment differences could be corrected using CAD tools to achieve an error of less than 1 degree for each patient.

The comparison of the sagittal plane angles between the live and cast models revealed no significant differences (p = 0.15), likely due to the large variability in alignment (x degrees to y degrees), as the live scans did not in the majority (78%) of cases align with the cast models. It was anticipated that only a small percentage of live scans would meet the one-degree target with their corresponding casts, as might be expected given the inherent alignment variability during traditional shape capture [6]. Although orthotists typically aim for a benchmark alignment (i.e., 90 degrees) a study by Ries et al reported that 60% of AFO casts deviate by more than 2 degrees from the benchmark alignment [6]. Our data shows a similar trend in that orthotists may not consistently achieve their alignment goals during the digital shape capture. The higher plantarflexion of live models compared to casted ones was likely due to challenges in controlling the talocrural joint during scanning. This major joint that connects the distal ends of the tibia and fibula to the proximal end of the talus is largely responsible for plantarflexion and dorsiflexion of the foot [28]. These findings suggest, that correcting sagittal-plane alignment may be necessary using live scanned models, similarly as is done by wedging in the traditional practice using physical casts [6]. However, it should be noted, that none of the casted models in this study needed alignment adjustment.

In this study, we corrected the live-scan sagittal-plane alignment to match the casted one. The purpose of this was two-fold. One was to facilitate shape comparison, and the second was to assess if the digital correction tools could achieve acceptable results, to which the answer is yes. The model pairs had alignments that were all (with the exception of one pair) within +/- one degree. Whether orthotists are targeting a neutral, dorsiflexed, or plantarflexed position, the results of this study show that CAD can be used to achieve the desired sagittal-plane alignment with high accuracy (<1 degree error).

Analysis of global shape differences revealed that the live scans exhibited a slightly larger volume compared to the cast models. These differences in overall volume are the accumulation of surface deviations (cloud-to-mesh distances) across the entire shape. Upon examining these deviations more closely, the average cloud-to-mesh distance across all models was found to be less than 0.5 mm. These surface deviations are likely to be clinically insignificant with respect to overall fit and function of the AFO [43]. Although limited research exists on AFOs, a study by Sanders et al. explored the impacts of socket size on the fit of transtibial prosthetic sockets [43]. The participants’ as prescribed sockets were enlarged or reduced by 1.8 mm, a change that was noted as relatively small which may have limited clinical impact. In the context of prosthetic sockets, changes of this magnitude typically correspond to very early stages of socket fit deterioration. While it is not a direct comparison, these changes are significantly greater than the average surface deviations observed in the AFO models. It can be concluded with confidence that the global shape differences are likely due to the compression of the lower limb by the casting tape during casting. Additionally, the pressure applied by the orthotist to the ankle and foot during this process may have further reduced the lower limb’s volume. This pressure helps to ensure that the ankle and foot are properly aligned across all anatomical planes.

The CSA analysis revealed negative CSA differences at both the distal and proximal ends of the model, consistent with the overall trend of more volume observed in the live scan. Again, this increased volume is consistent with clinical practice, as orthotists apply pressure in these areas to control ankle-foot alignment during casting [25]. Furthermore, the orthotists frequently had to apply downward pressure on the foot during the live scan to control ankle-foot alignment, which may have increased the volume at the distal end of the model. During traditional shape capture, more casting tape was applied to the ankle and foot compared to the leg, which also aligns with the results of the CSA analysis showing minimal CSA differences along the length of the leg. This additional casting tape helped the orthotists better stabilize the foot while the cast hardened.

**Fig 8:**
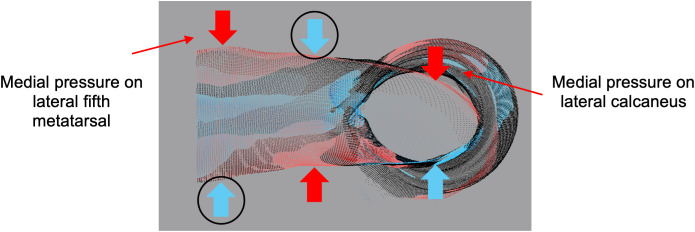
Modification map illustrating the three-point force system for correcting pronation of the foot. Pressure is applied laterally at the neck of the talus, serving as the center force in the three-point force system. Medial pressure is applied at the lateral calcaneus and lateral fifth metatarsal. Black circles indicate areas that deviate from the three-point force system.

Locally, the most significant shape differences occurred in areas of the foot as indicated by the modification maps. The reliefs observed along the dorsum of the foot and the front of the leg were expected, as clinicians use a cut strip to remove the cast once it has hardened. Although the cast is stapled back together, it tends to bulge along the cut strip. When examining the areas of relief and compression on the medial and lateral sides of the foot and heel, the observed trend aligns with the three-point force system for correcting ankle-foot alignment (sagittal, frontal, and transverse) (Fig 8) [44]. Pronation of the foot is corrected with medial pressure at the lateral calcaneus and fifth metatarsal, while supination is corrected with lateral pressure at the medial calcaneus and first metatarsal, with counterforces applied at the talus. Deviations from the three-point force system could be attributed to both the compression of the cast during traditional shape capture and the downward pressure applied to the foot during digital shape capture. This would expand the volume of the foot and exaggerate the shape differences, potentially leading to CMD thresholds that were not sensitive enough to capture the surface deviations between the live scan and cast.

The modification maps revealed that both compression and alignment (frontal and transverse) can affect the shape of the models obtained through traditional and digital shape capture methods. In traditional shape capture, compression helps the casting material closely adhere to the contours of the lower limb, ensuring an accurate representation of its shape [45]. The orthotists use the three-point pressure system to ensure proper alignment in all three anatomical planes [44]. The casts therefore represent a fully corrected position of the lower limb. Conversely, digital shape capture benefits from avoiding compression to ensure accuracy, precision, and patient comfort [21]. In this study, the live scans provide a detailed representation of the lower limb, corrected only in the sagittal plane. For all participants, the modification maps highlight the need to correct frontal and transverse plane alignment as well. This will likely need to be done digitally, as it would be challenging to perform a live scan while manipulating the subtalar joint. In working towards developing a fully digital workflow, an emphasis should be placed on alignment to ensure that the AFOs provides the intended support and correction. Misalignment in multiple anatomical planes may lead to tissue injuries and reduced gait efficiency [46]. Once proper alignment is achieved, compression can be adjusted using CAD design tools to modify the models as needed.

Analysis of CMD values showed that participants’ height, age, and foot length had comparable and moderate effects on shape differences, reflecting the relationship between foot length and children’s height and weight [47]. Consistent with previous findings, no significant differences were observed in the CMD values between AFO types and primary diagnosis groups [33]. All differences were under 1 mm further confirming that the results are likely clinically insignificant for AFO fit and function [43]. However, the medium-large effect sizes and lack of statistical significance suggest the need for a larger sample size in future work [39]. Interestingly, despite the relatively small sample size and the limited number of orthotists, significant differences and large effect sizes were detected for the CMD values. This suggests that the difference detected was likely to be a true effect rather than a result of random variability [39]. However, when comparing the means of the CMD values, the maximum observed difference was approximately 1 mm, which is still considered clinically insignificant [43]. It is important to point out that these results highlight that the design and fabrication of AFOs are influenced, at least in part, by the individual skills of the orthotists. There is a need for standardized protocols and targeted training to help minimize orthotist-dependent variability. Ensuring consistency in limb positioning, alignment, and scan quality are critical for effective and reliable use of 3D scanning in AFO fabrication. When comparing means of the CMD values for gender groups, all differences were less than 1 mm. However, the medium effect size and lack of statistical significance again suggest that the non-significant results could again be attributed to a small sample size [39]. For prescription groups, all the effect sizes were small with no statistical significance. Comparison of the means of the CMD values revealed that the differences were all less than 1 mm, further confirming that the results are likely clinically insignificant [43].

Overall, the orthotists reported similar satisfaction scores for both traditional and digital shape capture. No significant differences were observed in the ease of the process or the results, but a slight significance was observed in the time required. This is promising and underscores the potential of DT to enhance the design and fabrication of AFOs. Orthotist satisfaction scores were collected only after they had been trained on the scanning process. Further evaluation is needed to explore whether satisfaction or performance improves over time. As with any new technology, there is a learning curve before users feel completely comfortable and become optimally efficient with the process. The feedback from the orthotists revealed that participants often needed more instruction, as many were experiencing 3D scanning for the first time. However, this is expected to improve as orthotists, patients, and families become more familiar with the scanning process. Anecdotal feedback from participants indicated that the scanning experience was positive. Although this feedback was not part of a formal evaluation, it suggests a favourable response to the 3D scanning process. Future studies should assess participant scanning experience and satisfaction to better understand and optimize the scanning process. Orthotists found the setup for digital shape capture to be easy and quick. They also noted that their positioning during this process was more ergonomic compared to casting. While they were able to visualize ankle-foot alignment across all anatomical planes, they faced challenges in controlling alignment in the frontal and transverse planes. This was largely due to their inability to control the subtalar joint during scanning, as they were instructed to hold the patient’s foot distal to the metatarsal heads. The orthotists also noted that the developed 3D scanning process required two people, which differs from traditional shape capture methods where the orthotist independently casts the patient. Despite this, the time savings associated with digital design and fabrication may enhance efficiency and reduce the manual labor needed to produce the AFO, particularly because the traditional process is labour intensive during the latter stages of the fabrication process (i.e., rectification and moulding and finishing).

This study has several limitations. Although consistent with other research [9,29], the sample size is relatively small, particularly for the subgroup analysis. This limitation is primarily due to the short recruitment period. Future research should involve a larger and more clinically diverse population, particularly including patients who cannot be casted in a neutral position. Increasing the sample size would also facilitate a more comprehensive analysis of the influence of orthotists and participant characteristics on both traditional and digital shape capture methods. It is important to note that this study did not evaluate spasticity levels, a critical factor that can complicate scanning. Future research should include clinical measures of spasticity, such as the Ashworth Scale, to assess its impact on the 3D scanning process [48]. In this study, traditional shape capture was performed using a well-established casting process by orthotists at our rehab hospital. However, alternative methods may be standard in other clinical environments, which could influence generalizability. Additionally, only a single live scan was analyzed for each participant and future studies should include multiple scans to evaluate repeatability. While about half of the participants in this study required multiple scans before the orthotist was satisfied with the results, rescanning was typically quick and efficient. Further, plaster modifications occur during traditional fabrication methods to achieve the desired shape and volume of the cast [33]. While this study focused on comparing direct lower limb scans to unmodified casts, future research should include final modified casts to better quantify and inform the future digital design of AFOs. Specifically, this work should explore 3D alignment correction in clinical practice to help validate the functional outcomes achieved using the 3D scanning process presented in this study. Finally, future work should assess alignments in other planes. This might involve the use of reference points for 3D ankle measurements [49]. Similar to this study, digital tools could also be used to measure and correct alignment in these additional planes. However, the development of these methods should involve collaboration with orthotists to ensure the digital tools do not unintentionally alter the geometry of the ankle and foot. It is important to verify that these methods are adjusting the model in a way that mimics the biomechanics of the ankle and foot.

## Conclusion

This study uniquely compared scanned and casted models of pediatric AFOs. Through a data-driven and quantitative approach, the preliminary findings show the potential of using 3D scanning and CAD for AFO design and fabrication. By utilizing DT to quantitatively measure and adjust ankle-foot alignment, as well as to identify global and shape differences between models acquired using traditional and digital shape capture, this research supports the future advancement of digital orthotic design.
